# Impaired Pituitary Axes Following Traumatic Brain Injury

**DOI:** 10.3390/jcm4071463

**Published:** 2015-07-13

**Authors:** Robert A. Scranton, David S. Baskin

**Affiliations:** Department of Neurosurgery and the Kenneth R. Peak Brain and Pituitary Tumor Treatment Center, Houston Methodist Neurological Institute, 6560 Fannin St. Suite 944, Houston, TX 77030, USA; E-Mail: rascranton@houstonmethodist.org

**Keywords:** traumatic brain injury, hypopituitarism, head trauma, pituitary deficiency

## Abstract

Pituitary dysfunction following traumatic brain injury (TBI) is significant and rarely considered by clinicians. This topic has received much more attention in the last decade. The incidence of post TBI anterior pituitary dysfunction is around 30% acutely, and declines to around 20% by one year. Growth hormone and gonadotrophic hormones are the most common deficiencies seen after traumatic brain injury, but also the most likely to spontaneously recover. The majority of deficiencies present within the first year, but extreme delayed presentation has been reported. Information on posterior pituitary dysfunction is less reliable ranging from 3%–40% incidence but prospective data suggests a rate around 5%. The mechanism, risk factors, natural history, and long-term effect of treatment are poorly defined in the literature and limited by a lack of standardization. Post TBI pituitary dysfunction is an entity to recognize with significant clinical relevance. Secondary hypoadrenalism, hypothyroidism and central diabetes insipidus should be treated acutely while deficiencies in growth and gonadotrophic hormones should be initially observed.

## 1. Introduction

Traumatic brain injury (TBI) is a significant problem in both the developed and undeveloped world. The 2010 Center for Disease Control and Prevention (CDC) estimate of the rate of emergency department visits, hospitalizations, and deaths from TBI in the United States was 823.7 per 100,000, an increase from previous years [1]. Falls accounted for 40% of TBIs from 2006–2010 with over half of those in children. The annual cost of TBI in the United States has been estimated at $13.1 billion in 2013 dollars [2].

Hypopituitarism is an underdiagnosed sequela of TBI. It was previously thought to be rare, comprising 0.7% of cases of hypopituitarism [3], but is increasingly recognized [4,5]. The first report was in 1918 associated with a basilar skull fracture [6]. Early descriptions included autopsy data associated with infarction of the anterior pituitary [7,8,9,10]. Understanding the true incidence is difficult given that TBI is not considered by many practitioners in the evaluation of hypopituitarism, even amongst endocrinologists [5]. There is also a significant variability in the timing of presentation with post TBI hypopituitarism ranging from a few days to over forty years, though most cases present within the first year [11,12,13,14,15,16,17,18,19,20,21]. Pituitary dysfunction in cases presenting so remotely is usually a diagnosis of exclusion, creating a large degree of skepticism in attributing TBI as the etiology.

Ascertaining the rate of pituitary dysfunction following TBI from the literature is difficult given inherent variability in study design and methods [22]. Much of the available data is retrospective in nature with only a few prospective series; some even combine the two methods. Prospective series also vary in latency between injury and testing. The completeness of evaluation of the hypothalamic-pituitary axis also varies among reports with some excluding certain hormones and others only measuring static hormone levels rather than combining static assessments with provocative tests.

## 2. TBI Related Anterior Pituitary Dysfunction

Post TBI anterior pituitary dysfunction is reported between 28% and 80% [12,23,24,25,26,27,28,29,30,31]. This inconsistent data is felt to be a result of both selection and information bias within studies that do not allow for direct comparison. A higher quality twelve month prospective study of the rate of post TBI hypopituitarism reported a 33% incidence at three months and 23% at one year, with deficiencies being isolated, multiple or complete in 12%, 4%, and 6% respectively [26,32]. Another prospective study found anterior pituitary dysfunction at 3 and 12 months in 56% and 36% of patients, respectively [29]. Growth hormone deficiency is thought by many to be the most common post TBI pituitary disturbance [23,24,25,26,32,33,34,35], some purport gonadotrophic dysfunction or secondary hypoadrenalism to be more prevalent [5,12]. An overview of data from select studies is presented in Table 1.

**Table 1:** Summary of pituitary deficiencies in select studies.

Study Design	Overall Rate	Adrenal	Thyroid	Prolactin	Gonadal	Growth Hormone	Antidiuretic Hormone
Prospective, 45 patients Reported percent day1/day4 post TBI [31]	54%/70%	54%/70% (Serum cortisol <10 µg/dL)	Low fT4 5.5%/27.3% Low TSH 4.5%/15.9%	67%/77%	Females excluded. Low testosterone 82.1%/100%, LH 55.2%/58.6%, FSH 10.3%/37.9%	30.2%/2.3% (based on IGF-1)	No data
Prospective 50 patients median 12 days post TBI [36]	80%	16% (glucagon stimulation)	2% (fT4, TSH)	52%	80% (79% males low testosterone, 90% females low estradiol)	ITT 18% (peak GH <5 ng/mL), 16% (<3 ng/mL)	26%
Prospective 78 patients, reported 3/12 months post TBI [29]	56%/36%	19%/9% (short ACTH stimulation)	8%/3% (T3, fT4, and TSH)	3%/4%	32%/21% (FSH, LH, testosterone and estradiol)	9%/10% (GHRH + Arg stimulation)	No data
Prospective 70 patients reported 3/12 months post TBI [26,32]	32.8%/22.7%	8.5%/7.1% (AM cortisol and 24 h urine cortisol)	7.5%/5.7% (T3, fT4, TSH)	4.2%/5.7%	17%/11.4% (FSH, LH, testosterone, and estradiol)	21%/20% (GHRH + Arg stimulation)	9.2%/2.8%
Retrospective analysis of prospective database 102 TBI survivors median 17 months [12]	28.4%	12.7% (Glucagon stimulation or ITT)	0.98% (TSH, fT4; single patient with panhypopituitarism)	11.8%	11.8% (males only by testosterone and gonadotropin)	7.8% (by (glucagon stimulation, GHRH + Arg, or ITT)	No data
Prospective, 70 patients median 13 months post TBI [23]	68.6%	45.7% (AM cortisol) 7.1% (Short ACTH stimulation)	21.7% with any abnormality. (TSH 10%, fT4 8.6% and both 2.9%)	Increased in 6 males and 1 female (5 males and single female on PRL elevating drug)	None	14.6% (glucagon or l-DOPA stimulation)	No data
Retrospective and prospective. 22 patients median of 26 months post TBI [24]	36.4%	None (ITT)	4.5% (TRH stimulation, TSH, fT4)	62%	22.2% males (GHRH, but all with normal testosterone). 25% females (GHRH, LH and estradiol)	18.2% (ITT)	No data

TBI, traumatic brain injury; fT4, free thyroxine; TSH, thyroid-stimulating hormone; LH, luteinizing hormone; FSH, follicle-stimulating hormone; IGF-1, insulin-like growth factor-1; ITT, insulin tolerance test; GH, growth hormone; ACTH, adrenocorticotropic hormone; GHRH, growth-hormone-releasing hormone; Arg, arginine; AM, ante meridiem; PRL, prolactin; l-DOPA, l-3,4-dihydroxyphenylalanine; TRH, thyrotropin-releasing hormone.

### 2.1. Pathologic Mechanisms

The corticotrophic and thyrotrophic cells are located in the anterior pituitary ventrally and medially and supplied primarily by the short portal veins. The somatotrophic cells are located laterally with the majority of its vascular supply provided by the long portal veins [5,7,24,35,37]. Increased intracranial pressure and traumatic injury can occlude the long hypophyseal vessels traversing through the diphragma sella, while sparing the short portal veins arising below, sparring the medial anterior pituitary. Shock, cerebral vascular accidents and other low-flow states can cause similar injury. Traumatic transections of the stalk can have a unique influence on pituitary gland function. Hypothalamic signals may be disrupted but 70%–90% of flow to the portal system is from the superior hypophyseal artery via the long portal vein which may remain intact, sparing the gland from infarction and allowing a greater theoretical chance of long-term recovery [37,38,39].

Moderate and severe TBI may result in disruption of the blood brain barrier allowing the influx of systemic inflammatory cytokines such as IL-1β, IL-8, and Tumor necrosis factor α to name a few [40,41]. Neurons, glial cells and microglia also produce inflammatory cytokines and excitotoxcicty with glutamate release in response to trauma causing secondary injury [42,43,44]. More recently the complement pathway has been implicated in CNS damage following TBI and discussed as a potential therapeutic target [45]. The precise role that these factors play in post-traumatic hypopituitarism is unclear.

Anti-pituitary antibodies (APA) and antihypothalamus antibodies (AHA) has been demonstrated in patients with both idiopathic and autoimmune hypopituitarism [46,47,48,49,50]. The presence of APA has also been demonstrated in almost half of TBI patients in one study, with 46% of those positive for APA developing pituitary dysfunction at three years [51]. A series examining amateur boxers found antihypothalamus and antipituitary antibodies in 21% and 23%, respectively [52]. These series provide preliminary evidence that the pathophysiology of post-TBI hypopituitarism may involve autoimmunity in some cases.

### 2.2. Pituitary-Adrenal Axis

Olivecrona *et al.* prospectively studied morning cortisol levels in 45 (30 male/15 female) patients with severe TBI (Glasgow Coma Scale <8, GCS) and found them to be below 10 µg/dL in 54% and 70% of subjects on hospital days one and four respectively [31]. There was no correlation between GCS at presentation, intracranial pressure, or three-month mortality and functional outcome with serum cortisol level. This study did not include any provocative tests or further investigation into the cause of adrenal failure. In a series studying the acute changes following TBI at a median of twelve days, 50 consecutive patients with initial GCS of 3–13 underwent a glucagon stimulation test. Investigators reported 16% had peak cortisol responses below 450 nmol/L [36]. The cortisol deficiencies were unrelated to patient age, body mass index (BMI), or GCS at presentation. Another study reported post TBI secondary adrenal failure further out at three and twelve months as 19% and 9%, respectively utilizing the short adrenocorticotropic hormone (ACTH) stimulation test with synacthen [29]. Aimaretti and associates studied post TBI endocrine disturbances in a prospective manner in 100 patients, measuring morning serum cortisol and 24 h urine cortisol levels. They found secondary adrenal failure at a lower rate, 8.5% and 7.1% of patients at three and twelve months respectively [26,32]. A large study of 102 consecutive TBI survivors evaluated with both glucagon stimulation and insulin tolerance tests and found adrenal failure in 12.7% of participants when studied at a median of 17 months after TBI [12]. Lieberman *et al.* found morning cortisol below normal in 45.7% of 70 adults studied at a median of thirteen months post TBI, but adrenal failure in only 7% after cosyntropin stimulation [23]. The available data suggests that patients with post TBI secondary adrenal failure may recover function as time progresses. Despite some patients improving, vigilance is necessary as others may develop an endocrine disturbance in a delayed fashion [26,32].

The relatively high incidence of adrenal failure post TBI is of great clinical significance. Adrenal failure contributes to hypotension, hyponatremia, and hypoglycemia [11,53] and may occur in post TBI patients requiring more vasoactive medications [24,54,55,56]. Care must be taken prior to treating a trauma patient with hyponatremia and hypotension with stress doses of steroid supplementation. The patient may suffer from the syndrome of inappropriate antidiuretic hormone, systemic inflammatory response syndrome, or sepsis where high dose supplementation may be harmful, so careful assessment of all the hormone axes is required [57,58,59].

After basal cortisol levels suggest ACTH deficiency, usually with basal levels less than 7 µg/dL, a provocative test can be performed [30]. The low dose ACTH stimulation test with 1–2 µg ACTH confirms deficiency with a peak cortisol level less than 20 µg/dL or 497–500 nmol/L [60,61,62]. A number of technical considerations relating to this test can make proper administration difficult including limited availability of accurate 1-µg doses and the bolus administration not allowing for a steady state plasma concentration, amongst others [63,64,65]. The standard dose ACTH stimulation tests involve administering 250 µg ACTH with sampling 30–60 min after administration with peak cortisol response greater than 18 µg/dL ruling out adrenal insufficiency [63,66]. This test may be unreliable in acute secondary adrenal insufficiency when there is inadequate time for adrenal atrophy and a loss of responsiveness to such a high dose [67,68]. In the insulin tolerance test 0.15 U/kg insulin intravenous insulin is given to achieve glucose less than 40 mg/dL with a cortisol level less than 18 µg/dL confirming deficiency [12,69,70,71].

### 2.3. Pituitary-Thyroid Axis

The thyrotrophic cells, like the corticotrophic cells, are located ventrally and medially within the pituitary and share similar susceptibilities as previously discussed [24,35] with ischemic necrosis being particularly prominent [9,72]. Recommended screening for thyroid deficiency following TBI, regardless of severity, includes basal levels of free triiodothyronine (fT3), free thyroxine (fT4), and thyroid stimulating hormone (TSH) with further testing at three months and 12 months. Testing beyond 12 months is indicated if there are clinical signs of hypopituitarism [35]. Rates of secondary hypothyroidism range from 0.9%–8% [12,26,29,32].

Acutely, a small prospective study of 50 consecutive patients admitted to an intensive care unit (ICU) post TBI included testing TSH and fT4 on a median of day 12 (range 7–20). The study found one patient (2%) with secondary hypothyroidism [36]. In a series of 70 adults post TBI with a median testing time of 13 months, 21.7% of patients had an abnormality of TSH (10%), fT4 (8.6%), or both (2.9%) [23]. In this study there were 15 patients with low fT4 and TSH, only six underwent thyrotropin-releasing hormone (TRH) stimulation, with subnormal responses in half. TRH stimulation is performed with 0.5 mg intravenously with a normal response indicated by a peak TSH increase of 5–30 mU/L [23]. In Aimaretti’s prospective series of 100 TBI patients evaluated at three and 12 months post TBI, secondary hypothyroidism was diagnosed in 7.5% and 5.7% of patients respectively on the basis of fT3, fT4, and TSH [26,32]. Schneider *et al.* also prospectively investigated 78 patients at three and 12 months post TBI diagnosing secondary hypothyroidism (via T3, fT4, and TSH) in 8% and 3% of patients, respectively [29]. The authors also found a higher rate of secondary hypothyroidism initially with a decline at one year.

### 2.4. Prolactin

Studies addressing prolactin secretion dysfunction following traumatic brain injury are variable in their results [12,16,18,24,25,26,29,32,36]. This paucity of quality data may be the result of a perceived lack of clinical significance relative to other hormones such as cortisol, thyroid stimulating hormone and growth hormone [73].

In the hyperacute setting, 44 patients with severe traumatic brain injury (sTBI) defined as a Glascow Coma scale (GCS) ≤ 8 measured on day 1 and 4 showed elevated prolactin levels in 67% and 77% of patients, respectively [31]. Prolactin levels were not associated with increased mortality, unfavorable or favorable outcome, GCS, Marshall Grade, minimum cerebral perfusion pressure or maximum intracranial pressure at three months. Agha *et al*. studied 50 prospective patients with moderate to severe TBI as defined by GCS at a median of 12 days post injury finding hyperprolactinemia in 52% of patients [36]. Prospective data following TBI found hyperprolactinemia in 4.2% and 5.7% of patients at three and 12 months, respectively [18,26,32]. This largely agreed with a similar report finding three- and 12-month rates of 3% and 4%, respectively [29].

Kelly *et al.* found a high percentage (62%) of patients with elevated prolactin levels measured at a median of 26 months (range three months to 23 years) post TBI. No patient had an abnormal response to stimulation after testing with thyroid releasing hormone stimulation [24]. The United Kingdom Blast Injury Outcome Study of Armed Forces Personnel focused on non-penetrating blast induced TBI in 19 patients 2 to 48 months after injury and found two patients (10.5%) with hyperprolactinemia [16]. Most disturbances of prolactin levels post TBI will manifest as hyperprolactinemia, due to inhibition of transport of prolactin inhibitory factor down the pituitary stalk into the gland.

### 2.5. Pituitary-gonadal Axis

The pituitary gonadal axis was examined in detail after sTBI by Olivecrona *et al.* However, women were excluded because it was decided that there were too many confounding variables including pre- or post-menopausal states, exogenous hormones, and phase of the menstrual cycle [31]. The authors found low serum testosterone in 82.1% of men at day one and 100% by day four. Similar deficiency was found for day one and four luteinizing hormone (LH) (55.2%/58.6%) and follicle stimulating hormone (FSH) (10.3%/37.9%). Elevations of day one and four LH (6.9%/6.9%) and FSH (6.9%/3.4%) were observed. Interestingly, lower levels of total and free calculated testosterone on day one as well as LH and FSH correlated with increased survival at three months. Wagner *et al.* also reported early suppression of the pituitary-gonadal axis after TBI with low LH in 83%, FSH in 63%, and testosterone in 100% of men and low estradiol in 43% of premenopausal women [21]. Klose *et al.* reported 68% of patients had hypogonadotropic hypogonadism after acute TBI [33]. In the acute phase Agha *et al*. reported gonadotropin deficiency in 80% of patients unrelated to the presence of prolactin [36]. Chronically at a median of 17 months post TBI gonadotrophic deficiencies were observed in 11.8% [12]. In a year-long study gonadal failure was seen in 17% and 11.4% of patients at three and 12 months, respectively [26,32]. This early, high percentage of patients with gonadotrophic failure with a tendency to improve was confirmed by Schneider *et al.* who saw failure in 32% and 21% at three and 12 months, respectively [29].

Studies outside the TBI literature suggest that suppression of the hypothalamic gonadal axis may be an adaptive feature to help the body cope with critical illness and trauma and the ensuing catabolic state [74,75,76,77,78]. The TBI literature also illustrates a suppression of the hypothalamic gonadal axis [12,14,26,29,31,32,33,36], however in cases of sTBI there may be a smaller degree of suppression with poorer long-term prognosis [31]. The role of sex hormone replacement after traumatic brain injury is unclear at this time [36].

### 2.6. Growth Hormone Axis

Insulin-like growth factor 1 (IGF-1) levels are often used as a surrogate marker for growth hormone deficiency (GHD), however there may be patients with severe GHD and normal IGF-1 levels [35,79,80,81,82,83,84,85]. It is recommended that provocative testing be performed to confirm the diagnosis of GHD with tests such as the insulin tolerance test (ITT), glucagon stimulation or growth-hormone-releasing hormone (GHRH )+ arginine or secretalogues [86,87,88,89,90,91,92]. The ITT test is normal with peak growth hormone (GH) greater than 5 µg/L and indicative of severe deficiency when less than 3 µg/L [23,93,94]. Some caution against the use of the ITT in patients with coronary heart disease, seizure disorder, or those at risk of seizures such as TBI patients [95,96,97]. Notably, Kopczak *et al.* performed the ITT in 41 patients with traumatic brain injury and another 15 patients with subarachnoid hemorrhage, a high-risk population, without a single seizure observed [98]. The ITT and glucagon tests act on the hypothalamus while GHRH acts on the somatotrophs of the pituitary creating the potential for a false negative with the latter. The GHRH + arginine test is performed with suggested deficiency values varying between 4–11 µg/L depending on BMI [93]. Ghigo *et al.* define severe deficiency as peak less than 9 µg/L and less than 10 µg/L with the GHRH + GH releasing peptid-6 test [89]. Unfortunately GHRH has limited availability in the US and the possibility of false negatives with hypothalamic dysfunction make it of limited utility after TBI. Glucagon is more readily available and acts on the hypothalamus with suggested deficiency cut-off values after stimulation less than 2.5–3 µg/L [91,99,100,101,102,103]. Glucagon stimulation may be the test of choice in evaluating TBI induced GHD [91]. Growth hormone deficiency testing should not be undertaken after TBI until other hormone deficiencies have been treated [91,93].

In a study measuring (GH) levels acutely at day 1 and 4 after sTBI there was tremendous variation with no relation to diurnal patterns [31]. However, in the same patients IGF-1 levels were low in 30.2% on day 1 and 2.3% on day 4 with 7.0% of patients with high levels on day 4. There was no statistically significant relationship to GCS, Marshall Grade, intracranial pressure (ICP) or three-month mortality or Glasgow outcome score (GOS). In the series of 101 TBI patients by Wagner *et al.*, there was an initial decline of GH followed by normalization or slight increase and a trend of decline in IGF-1 levels acutely with recovery over the 10 day study period; 77% of the patients had at least one low IGF-1 level [21]. Acutely high GH with low IGF-1 quickly changing to normal GH and elevated IGF-1 levels after trauma has been supported by multiple studies [21,31,104]. A proposed mechanism for this includes peripheral GH resistance in critically ill patients [105]. The first two studies presented by Olivecrona and Wagner did not include provocative testing, the significance of these values and whether they represent a true deficiency cannot be determined. In a study of 50 consecutive patients with moderate to severe TBI utilizing the ITT, 18% of patients had peak GH less than 5 ng/mL and 6% had a peak less than 3 ng/mL [36].

Examination of TBI patients with GH, IGF-1 levels and GHRH + arginine showed 3- and 12-month deficiency rates of 21% and 20%, respectively [26,32] in one study and 9% and 10% in another [29]. Lieberman *et al.* reported GH deficiency in 14.6% of patients at a median of 13 months post TBI [23] and another study reported 7.8% at a median of 17 months [12], both confirmed their findings by provocation tests.

The somatotrophic cells are supplied by the long portal vessels, located in the wings of the pituitary gland, and exquisitely sensitive to damage [24,35]. Growth hormone deficiency is thought to be the most common post TBI endocrine disturbance after three months [25,26,33,34] and is the most likely to spontaneously recover. Chronic GHD is known to affect long-term quality of life, mood, and lipid profile [93,106,107]. Some reports assert that GHD is associated with increased mortality [108,109,110,111,112], however this is disputed [113,114] and to what degree these reports apply to acquired GHD secondary to TBI is unknown [91]. The role of growth hormone replacement in the acute setting is unclear and may not be necessary.

## 3. TBI Related Posterior Pituitary Dysfunction

### Antidiuretic Hormone

Injury or dysfunction of the posterior pituitary is more clinically apparent than anterior pituitary dysfunction. Patients presenting with central diabetes insipidus (DI) or decreased free water excretion should be investigated for pituitary or hypothalamic damage [37,115]. If the DI lasts for several months, in most cases damage will have occurred either to the pituitary stalk or hypothalamus, as removal of the posterior pituitary itself does not produce permanent diabetes insipidus. A systematic review of hypernatremia after traumatic brain injury found an incidence between 16% and 40% [116] however this may not represent the incidence of DI given the heterogeneous methods of investigation the meta analysis is drawn from.

In the acute post TBI phase, at a median of 12 days post injury, 26% of TBI patients had DI which was unrelated to GCS and Marshall Grade and occurred independently of ACTH or GH deficiencies [36]. The prospective study by Aimaretti *et al.* reported DI in 4.2% and 2.8% of patients at three and 12 months, respectively [26,32]. Diabetes insipidus was reported in 14% of 50 TBI patients studied between 12 and 64 months post injury [25]. In an analysis of the literature and 367 cases of posttraumatic pituitary dysfunction, DI was present in 30.6% [5]. Post TBI DI has been studied recently with 102 patients reporting DI in 21.2% acutely and 7% by water deprivation test chronically at a median of 17 months [117]. Acute onset DI may recover long-term [14,18,117,118], however delayed onset after TBI can indicate stalk or hypothalamic damage and require life-long replacement [119].

## 4. Conclusions

Traumatic brain injury is a persistent global problem manifesting from a variety of causes including but not limited to: abuse, vehicle collisions, armed conflict, recreational accidents, sporting events and falls. CDC data shows that in the United States the leading cause of TBI is a fall, disproportionately affecting those under 14 and over 65, and also the leading cause of TBI related hospitalization and death [1]. Vehicle collisions are now the third leading cause of TBI and second leading cause of TBI related deaths.

The exact mechanism of TBI induced pituitary dysfunction is poorly understood but several observations have been made including anterior lobe infarction and necrosis from direct trauma or vascular injury, posterior lobe hemorrhage and stalk laceration [5,37,38,39,72]. Secondary injury from cytokines, excitotoxicity, complement, and APA and AHA may play a role but require further investigation especially given the possibility for intervention compared with direct trauma or vascular injury.

Consensus guidelines (2005) recommend screening all TBI patients with moderate or severe head injuries, or those with any severity presenting with a clinical picture compatible with hypopituitarism [35]. Basal hormone testing on any hospitalized patient with hypotension or hyponatremia and testing at three and 12 months regardless of TBI severity should be performed. Patients presenting with signs of hypopituitarism, beyond 12 months should still undergo evaluation. Basal testing includes: morning cortisol, fT3, fT4, TSH, IGF-1, FSH, LH, testosterone (males), estradiol (females), prolactin, and a 24 h urine collection for urinary free cortisol. Patients with polyuria should also have serum sodium and osmolality checked. Deficiencies must be confirmed with provocative testing. Class I evidence is unavailable regarding hormone replacement after acute TBI but secondary adrenal insufficiency, secondary thyroid insufficiency, diabetes insipidus and panhypopituitarism should be treated immediately. Growth hormone deficiency and secondary hypogonadism has a high degree of spontaneous resolution and should not be replaced until other deficiencies are appropriately managed.

Standardized definitions of TBI, deficiencies and adequate replacement are needed. Prospective studies should follow patients from the time of injury to death and examine quality of life parameters, productivity, and neuropsychometrics, and how they relate to the various pituitary deficiencies.
